# Evolution of nuchal glands, unusual defensive organs of Asian natricine snakes (Serpentes: Colubridae), inferred from a molecular phylogeny

**DOI:** 10.1002/ece3.4497

**Published:** 2018-09-21

**Authors:** Hirohiko Takeuchi, Alan H. Savitzky, Li Ding, Anslem de Silva, Indraneil Das, Tao Thien Nguyen, Tein‐Shun Tsai, Teppei Jono, Guang‐Xiang Zhu, Dharshani Mahaulpatha, Yezhong Tang, Akira Mori

**Affiliations:** ^1^ Seto Marine Biological Laboratory Field Science Education and Research Center Kyoto University Shirahama Japan; ^2^ Department of Biology Utah State University Logan Utah; ^3^ Chengdu Institute of Biology Chinese Academy of Sciences Chengdu China; ^4^ Gampola Sri Lanka; ^5^ Institute of Biodiversity and Environmental Conservation University Malaysia Sarawak Sarawak Malaysia; ^6^ Vietnam National Museum of Nature Vietnam Academy of Science and Technology Hanoi Vietnam; ^7^ Graduate University of Science and Technology Vietnam Academy of Science and Technology Hanoi Vietnam; ^8^ Department of Biological Science and Technology National Pingtung University of Science and Technology Neipu Township Taiwan; ^9^ College of Life Science Sichuan Agricultural University Ya'an China; ^10^ University of Sri Jayewardenepura Nugegoda Sri Lanka; ^11^ Department of Zoology Graduate School of Science Kyoto University Kyoto Japan; ^12^ Present address: College of Bioresource Science Nihon University Fujisawa Kanagawa Japan; ^13^ Present address: Tropical Biosphere Research Center University of the Ryukyus Nishihara Okinawa Japan

**Keywords:** *Balanophis*, *Macropisthodon*, molecular phylogenetics, Natricinae, nuchal glands, *Rhabdophis*

## Abstract

A large body of evidence indicates that evolutionary innovations of novel organs have facilitated the subsequent diversification of species. Investigation of the evolutionary history of such organs should provide important clues for understanding the basis for species diversification. An Asian natricine snake, *Rhabdophis tigrinus*, possesses a series of unusual organs, called nuchal glands, which contain cardiotonic steroid toxins known as bufadienolides. *Rhabdophis tigrinus* sequesters bufadienolides from its toad prey and stores them in the nuchal glands as a defensive mechanism. Among more than 3,500 species of snakes, only 17 Asian natricine species are known to possess nuchal glands or their homologues. These 17 species belong to three nominal genera, *Balanophis*,* Macropisthodon*, and *Rhabdophis*. In *Macropisthodon* and *Rhabdophis*, however, species without nuchal glands also exist. To infer the evolutionary history of the nuchal glands, we investigated the molecular phylogenetic relationships among Asian natricine species with and without nuchal glands, based on variations in partial sequences of Mt‐CYB, Cmos, and RAG1 (total 2,767 bp). Results show that all species with nuchal glands belong to a single clade (NGC). Therefore, we infer that the common ancestor of this clade possessed nuchal glands with no independent origins of the glands within the members. Our results also imply that some species have secondarily lost the glands. Given the estimated divergence time of related species, the ancestor of the nuchal gland clade emerged 19.18 mya. Our study shows that nuchal glands are fruitful subjects for exploring the evolution of novel organs. In addition, our analysis indicates that reevaluation of the taxonomic status of the genera *Balanophis* and *Macropisthodon* is required. We propose to assign all species belonging to the NGC to the genus *Rhabdophis*, pending further study.

## 1 INTRODUCTION

In the 20th Century, many biologists were focused on commonalities among taxa, as represented by studies using model organisms (Alberts et al., 2008). On the other hand, appreciating the diversity of life and its evolutionary origins has been another essential pursuit in biology (Rosenzweig, 1995; Whittaker, 1972). Because evolution of novel phenotypic characters, such as wings of birds and mammary glands of mammals, can facilitate the diversification of a lineage (Wagner & Lynch, 2010), investigation of the evolutionary history of such novel characters can provide basic information that clarifies the processes underlying species diversification.

Snakes (Serpentes) comprise a distinct monophyletic taxon within the Squamata (Pyron, Burbrink, & Wiens, 2013), including over 3,500 species that are distributed on all continents except Antarctica (Wallach, Williams, & Boundy, 2014). In spite of their seemingly uniform appearance, snakes exhibit prominent morphological and ecological diversity (Greene, 1997; Lillywhite, 2014) and have often evolved novel organs that serve particular ecological functions. A well‐known example of a novel defensive structure is the rattle of rattlesnakes, which is used to warn potential predators of the snakes’ venomous bite (Greene, 1997). The rattle evolved once in the ancestor of extant rattlesnakes (Castoe & Parkinson, 2006; Greene, 1997), and it has been lost secondarily in some island populations, where selection for defense is reduced in the absence of mammalian predators (Martins, Arnaud, & Murillo‐Quero, 2008; Rowe, Farrell, & May, 2002).

**Figure 1:**
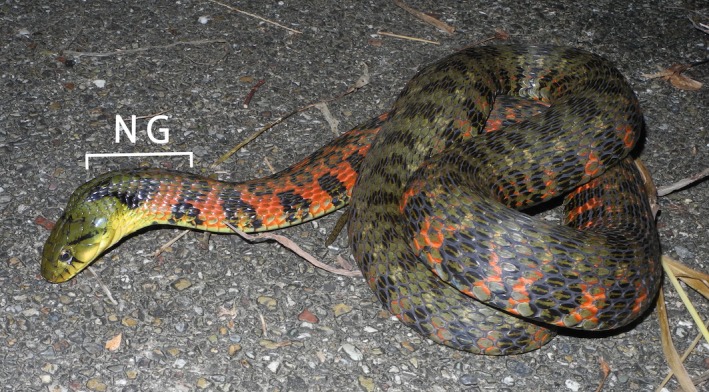
The snake, *Rhabdophis tigrinus*, in a defensive posture is directing the nuchal glands (NG) toward a perceived threat

The nuchal gland system is another example of a novel defensive structure that has evolved in snakes (Mori et al., 2012). Nuchal glands were originally described in a Japanese natricine snake, *Rhabdophis tigrinus* (Figure 1; Nakamura, 1935). The organs, which superficially resemble secretory structures, are embedded in the dermal layer of the dorsal skin of the neck. The nuchal glands of *R. tigrinus* contain cardiotonic steroid toxins known as bufadienolides (Hutchinson et al., 2007), which are sequestered from toads consumed as prey and can be redeployed as a defensive mechanism (Hutchinson et al., 2007). The glands of some other species also contain bufadienolides (Mori et al., unpublished). Ontogenetically, the nuchal glands are of mesodermal origin (Fukada, 1958; Mori et al., 2012), which is different from any other skin glands of terrestrial vertebrates, all of which arise from ectoderm (Savitzky et al., 2012). The glands lack a secretory epithelium and consist of a homogeneous population of fluid‐filled cells surrounding a dense aggregation of capillaries. There is no central lumen or duct, and the glands simply rupture through the skin to expel their fluid contents when the snake is under predatory attack (Mori et al., 2012).

Nuchal glands and the structurally similar nucho‐dorsal glands (which extend the full length of the body; Smith, 1938) are currently known in 17 species of Asian Natricinae (Mori et al., 2012; Mori, Jono, Ding, et al., 2016). Hereafter, we refer to all such structures as nuchal glands, for simplicity. No other animals have been reported to possess organs similar in their structural details to the nuchal glands. The 17 species that possess such glands belong to three nominal genera, *Balanophis*,* Macropisthodon*, and *Rhabdophis*. Interestingly, *Macropisthodon* and *Rhabdophis* also include species that do not have nuchal glands (Table 1). This distribution might indicate the occurrence of (a) multiple independent origins of these unusual organs, (b) their secondary loss, and/or (c) improper generic assignment of some species.

**Table 1:** A species list for the three nominal genera, *Balanophis*,* Macropisthodon*, and *Rhabdophis*

Species	Glands	Source
***Balanophis ceylonensis***	P	Smith (1938)
***Macropisthodon flaviceps***	A/P	Smith (1938)
***M. plumbicolor***	P	Mori, Jono, Takeuchi, Ding et al. (2016) and Smith (1938)
*M. rhodomelas*	P	Smith (1938)
***M. rudis***	A	Smith (1938) and Takeuchi and Mori (2012)
***Rhabdophis adleri***	P	Mori, Jono, Ding et al. (2016)
*R. akraios*	U	Doria, Petri, Bellati, Tiso and Pistarino (2013)
*R. angelii*	U	Mori et al. (2012)
*R. auriculatus*	U	Mori et al. (2012)
*R. barbouri*	U	Mori et al. (2012)
***R. callichromus***	P	Mori et al. (2012) and Smith (1938)
*R. chrysargoides*	U	Mori et al. (2012)
***R. chrysargos***	A	Smith (1938)
***R. conspicillatus***	A	Mori, Jono, Takeuchi and Das (2016)
***R. formosanus***	P	Mori et al. (2012) and Takeuchi, Ota, Oh and Hikida (2012)
***R. guandongensis***	U	Zhu, Wang, Takeuchi and Zhao (2014)
***R. himalayanus***	P	Smith (1938)
***R. lateralis***	P	Mori et al. (2012) and Takeuchi et al. (2012)
***R. leonardi***	P	Mori et al. (2012)
*R. lineatus*	U	Mori et al. (2012)
***R. murudensis***	A/P	Mori et al. (2012), Smith (1938), and Steubing and Lian (2002)
***R. nigrocinctus***	P	Smith (1938)
***R. nuchalis***	P	Mori et al. (2012), Mori, Jono, Takeuchi, Ding et al. (2016), and Smith (1938)
***R. pentasupralabialis***	P	Mori et al. (2012) and Mori, Jono, Takeuchi, Ding et al. (2016)
*R. spilogaster*	A	Smith (1938)
***R. subminiatus***	P	Smith (1938)
***R. swinhonis***	A/P	Mao and Chang (1999) and Hsiang, Li and Yang (2009)
***R. tigrinus***	P	Mori et al. (2012) and Nakamura (1935)

Note

Species included in the analyses of this study are shown by bold. P, A, and U indicate present, absent, and unknown, respectively. Our study strongly suggests that *Balanophis* and *Macropisthodon,* except *M. rudis,* belong to *Rhabdophis*.

To infer the evolutionary history of the nuchal glands, we investigated the molecular phylogenetic relationships among Eurasian natricine species, including all but one of the species that have hitherto been reported to possess such glands (Table 1). Our phylogeny is based on partial sequences of the oocyte maturation factor Mos (Cmos) gene, the recombination‐activating gene 1 (RAG1), and the mitochondrial cytochrome b (MT‐CYB) gene, for a total of 2.7 kbp. Several recent phylogenetic studies of snakes have either focused on or included a number of Asian natricine species (Figueroa, Mckelvy, Grismer, Bell, & Lailvaux, 2016; Guo et al., 2012, 2014; Pyron, Kandambi et al., 2013). However, no previous study has addressed the evolution of the nuchal glands. Furthermore, our sampling of species and populations of *Macropisthodon* and *Rhabdophis* is much greater than that of previous studies.

Specifically, our main purpose was to answer three questions: (a) Have the nuchal glands originated only once, or have they arisen multiple times independently among natricine snakes? (b) Do the species of *Macropisthodon* and *Rhabdophis* that lack such glands represent the secondary loss of those structures? (c) Are any of the species lacking nuchal glands incorrectly assigned to *Macropisthodon* or *Rhabdophis*?

## 2 MATERIALS AND METHODS

A total of 122 sequences of natricine snakes and three sequences of outgroup taxa were used for phylogenetic analyses (Appendix 1). Of those, 54 sequences were obtained from GenBank. Because our preliminary analysis suggested that the sequence data for *Rhabdophis adleri* registered in GenBank were incorrectly identified, we did not use the GenBank data for that species. The other 68 sequences were obtained by the following methods.

In each sample, total DNA was extracted from liver, skeletal muscle, or tail tips, which had been preserved in 99.5% ethanol or in freezers, using the DNeasy Tissue Kit (Qiagen). The Cmos, RAG1, and MT‐CYB regions were amplified with a PCR System GeneAmp 2700 Thermal Cycler (Applied Biosystems), using an Ex Taq Polymerase Kit (Takara Bio Inc.) and primers S77/S78 for Cmos (Lawson, Slowinski, Crother, & Burbrink, 2005), R13/R18 for RAG1 (Groth & Barrowchlough, 1999), and L14910/H16064 for MT‐CYB (Burbrink, Lawson, & Slowinski, 2000). The thermocycling schedule for the polymerase chain reaction (PCR) was identical to that described by these previous studies. Before sequencing, unincorporated primers were removed from the PCR products using polyethylene glycol precipitation. Cycle sequencing reactions were performed with the Big Dye Terminator Cycle Sequence Ready Reaction Kit, version 3.1 (Applied Biosystems), using the same primers as for PCR. The samples purified by ethanol precipitation were sequenced with a 3130xl Genetic Analyzer (Applied Biosystems). All fragments were sequenced for both forward and reverse sense. We assembled them using the GAP 4 program (Staden, 1996).

Using CLUSTAL X (Thompson, Gibson, Plewniak, Jeanmougin, & Higgins, 1997), 125 sequences were aligned. Identical sequences from different specimens were treated as single units so that 114 sequences were recognized. To infer the phylogeny, we employed Maximum Likelihood (ML) using combined sequences (Cmos + RAG1 + MT‐CYB) and Bayesian inference (BI) using the sequence of mitochondrial DNA (MT‐CYB). For both data sets, the most appropriate pattern of sequence evolution was selected by applying the Bayesian Information Criterion (BIC; Schwarz, 1978), using MEGA5 (Tamura et al., 2011). We set the rate categories of discrete gamma rate heterogeneity as eight for ML and BI. Reliability of the ML tree was assessed by calculating bootstrap probability (BP; Felsenstein, 1985), with 1,000 replications. The BI tree was constructed using BEAST version 1.8 (Drummond & Rambaut, 2007), employing a single Markov chain Monte Carlo (MCMC) run for 50 million generations, sampled every 1,000 generations, and excluding the first 5 million generations as burn‐in. Convergence of the chains to the stationary distribution was checked by visual inspection, using TRACER version 1.6 (Rambaut, Suchard, Xie, & Drummond, 2007).

To estimate divergence times, we employed Bayesian relaxed‐clock dating, using BEAST version 1.8. Because no fossils of *Balanophis*,* Macropisthodon*, or *Rhabdophis* are known, we set the following calibration points: 30 Mya (*SD* = 0.115) at the crown of natricine snakes, 22 Mya (*SD* = 0.15) at the crown of the genus *Natrix*, and 16 Mya (*SD* = 0.15) at the crown of the genus *Thamnophis* (Guo et al., 2012).

## 3 RESULTS

**Figure 2:**
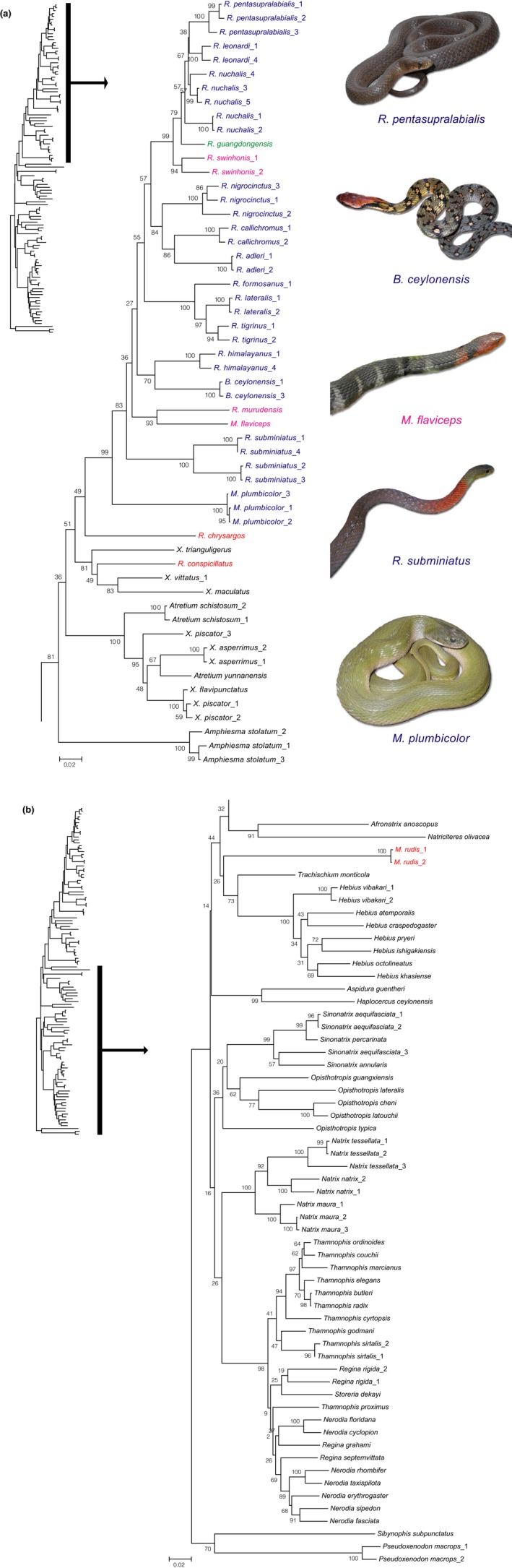
Maximum likelihood tree (−In *L* = −35078.3994) based on the combined sequence data of the MT‐CYB, Cmos, and RAG1 genes under GTR + G + I. Bootstrap probabilities are provided at each node. Numerals following scientific names indicate individual codes (see Appendix 1). Status of nuchal or nucho‐dorsal glands of our three focal genera (*Rhabdophis, Macropisthodon, and Balanophis*) is indicated by blue (present), red (absent), purple (present/absent), and green (unknown; see also Table 1). The photographs have been digitally modified for clarity. Photograph of *Balanophis ceylonensis* by Udaya Chanaka

**Figure 3:**
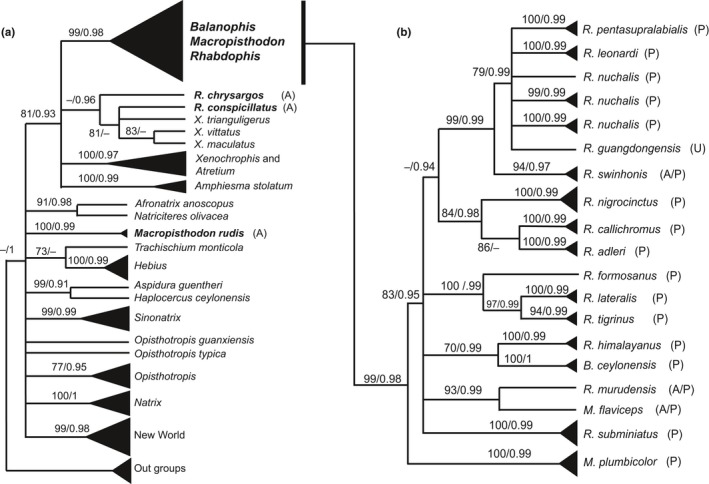
Consensus tree based on ML and Bl trees. Bootstrap probabilities (BP) from the maximum likelihood tree (left) and posterior probabilities (PP) from Bayesian inference (right) are shown at each node (shown only BP ≥ 70% and PP ≥ 0.90). (a) All Natricinae included in our analysis. Species of our three focal genera (*Rhabdophis*,* Macropisthodon*, and *Balanophis*) are indicated in bold. (b) Phylogenetic relationships among the nuchal gland clade. For the three focal genera, P, A, and U after the OTU indicate present, absent, or unknown condition, respectively, of nuchal or nucho‐dorsal glands (see also Table 1)

The final alignment of three gene fragments consisted of 2,767 aligned base pairs. Of those, 787–1,149 bp were from MT‐CYB (114 taxa), 259–689 bp were from Cmos (86 taxa), and 855–929 bp were from RAG1 (21 taxa). The most appropriate model under the BIC was the GTR + G + I model for the data sets of both the ML and BI trees. The ML and BI trees were almost identical in topology. The ML tree (−In *L* = −35078.3994) is shown in Figure 2. A consensus tree from the ML and BI analyses is shown in Figure 3, along with the BP values from ML and the posterior probability (PP) value from BI at each node (shown only for BP ≥ 70% in ML and PP ≥ 0.90 in BI). The main difference between the ML and BI trees is the status of *Rhabdophis chrysargos*. Unlike the ML tree, the Bl tree supported monophyly of *R. chrysargos* + *R. conspicillatus* + 3 species of *Xenochrophis* (Figure 3a).

Monophyly of Natricinae was strongly supported by the PP value. Within this subfamily, monophyly of the New World taxa (the Thamnophiini), and the Old World taxa *Natrix*,* Sinonatrix*,* Hebius*, and *Amphiesma *+* Xenochrophis *+* Atretium *+* Rhabdophis* + *Macropisth odon* (except *M. rudis*) + *Balanophis* clades were highly supported. Of the latter clade, a subclade of *Rhabdophis* (except *R. chrysargos* and *R. conspicillatus*) + *Macropisthodon* (except *M. rudis*) + *Balanophis* was separated from the remainder with strong support (Figure 2b). The average estimated divergence time of this subclade was 19.18 Mya (16.28–22.16 in 95% credible ranges). Hereafter, we refer to this subclade as the nuchal gland clade (NGC). Within this clade, *Macropisthodon plumbicolor* first diverged from the other species. The latter include *Rhabdophis subminiatus, R. murudensis* + *Macropisthodon flaviceps*,* R. himalayanus *+* Balanophis ceylonensis, R. tigrinus* + *R. lateralis* + *R. formosanus*, and a large group including *R. adleri* + *R. callichromus + R. nigrocinctus + R. swinhonis* + *R. guangdongensis* + *R. nuchalis* + *R. leonardi* + *R. pentasupralabialis* (with >90% support in BP and/or 0.9 in PP). The latter clade comprises two subclades: *R. adleri* + *R. callichromus + R. nigrocinctus* and *R. swinhonis* + *R. guangdongensis* + *R. nuchalis* + *R. leonardi* + *R. pentasupralabialis*. Several nominal species exhibit substantial population structuring. *Rhabdophis subminiatus* exhibits strong differentiation between Laos/Vietnam and Thailand samples, and *R. nuchalis* consists of a number of population segments and is paraphyletic with respect to both *R. leonardi* and *R. pentasupralabialis*.

## 4 DISCUSSION

Although differing in some details, recent molecular phylogenetic analyses of the Natricinae (Figueroa et al., 2016; Guo et al., 2012, 2014; Pyron, Burbrink et al., 2013; Pyron, Kandambi et al., 2013), including ours, agree on the general pattern of relationships among the major lineages. A basal dichotomy separates the subfamily into two major clades. One includes the entire North American natricine fauna (the Thamnophiini) and its sister group, the Eurasian genus *Natrix*. Those two, in turn, are sister to a clade containing the Asian genera *Opisthotropis* and *Sinonatrix*. A clade containing two endemic Sri Lankan genera, *Aspidura* and *Haplocerus*, is variously recovered as sister to this North American–Eurasian clade (Pyron, Burbrink et al., 2013; Pyron, Kandambi et al., 2013) or as the most basal branch of the natricine clade (our study, but with weak support).

The other major clade of natricines is almost entirely Asian, the sole exception being a monophyletic group of three African genera (*Afronatrix, Natriciteres,* and *Lycognathophis*, the latter not included in our analysis). The African clade is variously recovered as sister to, or embedded within, the much larger Asian radiation. The relationships among the Asian taxa display varying topologies among recent analyses, as taxon sampling within this group has improved. Consistent with other recent studies (Guo et al., 2014), we recover a monophyletic genus *Hebius*, distant from *Amphiesma stolatum*, as well as a polyphyletic *Xenochrophis*, some related to *Atretium* and others close to *Rhabdophis* and *Macropisthodon*. These results engender confidence in our analysis of the relationships within the NGC.

### 4.1 Evolution of the nuchal glands

Our results show that all species that possess nuchal glands belong to a single, strongly supported clade (NGC). Therefore, based on the principle of parsimony, we infer that the common ancestor of this clade possessed nuchal glands. We find no evidence of multiple, independent origins of the glands. Thus, interspecific differences in the distribution and morphology of the glands, such as the occurrence of nucho‐dorsal glands along the entire length of the body in *M. plumbicolor* and several species of *Rhabdophis* (Mori, Jono, Ding et al., 2016; Mori, Jono, Takeuchi, & Das, 2016; Smith, 1938) and the presence of elongate, nonsacculated glands accompanied by scaleless areas of skin in *M. rhodomelas* (not included in our analysis), *M. flaviceps*, and *B. ceylonensis* (Smith, 1938), are considered to represent alternative morphologies that arose after a single evolutionary origin of the nuchal gland system. Further study of the morphological details is needed to clarify the process of glandular diversification within this clade.

Among species currently included in *Rhabdophis* and *Macropisthodon*,* R. chrysargos*,* R. conspicillatus*, and *M. rudis* have been reported to lack nuchal glands (Table 1; Mori et al., 2012; Mori, Jono, Takeuchi, & Das, 2016). *Macropisthodon rudis* is only distantly related to the NGC (see below), and *R. conspicillatus* and *R. chrysargos* also belong to clades outside the NGC. Thus, the absence of the nuchal glands in these species does not constitute secondary loss. Rather, it appears that they have simply retained the ancestral condition of the absence of integumentary defensive glands.

*Rhabdophis swinhonis* has been reported to lack nuchal glands (Table 1; Mao & Chang, 1999). However, in contrast to *R. conspicill atus* and *R. chrysargos*, our analysis shows that this species occupies a position within the NGC. This strongly suggests that *R. swinhonis* has secondarily lost the nuchal glands. However, Hsiang, Li, and Yang (2009) noted the presence of nuchal glands in this species. If both observations are correct, there are two possible interpretations: either the occurrence of intraspecific variation or the presence of two distinct but cryptic species. Whichever is true, the deeply nested position of *R. swinhonis* within the NGC implies the recent or ongoing secondary loss of the glands in at least some populations.

Intraspecific variation in the presence of the nuchal glands also has been described in *R. murudensis* and *M. flaviceps* (Table 1; Smith, 1938; Mori et al., 2012). In our analysis, both species are recovered within the NGC. Therefore, as with *R. swinhonis*, the nuchal glands of *R. murudensis* and *M. flaviceps*, if accurately described in the literature, might be in a transitional stage of secondary loss or these nominal species may contain closely related cryptic species.

We estimate that the common ancestor of the NGC arose 19.18 Mya. This is only slightly later than the date of 23–24 Mya shown by Guo et al. (2012, Figure 2) for the origin of *Rhabdophis*, suggesting that nuchal glands arose at or soon after the origin of this genus.

### 4.2 Taxonomy

Our analysis requires a reevaluation of the taxonomic status of the genera *Balanophis* and *Macropisthodon*. The validity of the monotypic genus *Balanophis* (Smith, 1938) has been controversial. Malnate (1960) recognized the species as *Rhabdophis ceylonensis*, and McDowell (1961) supported his position. Figueroa et al. (2016) found the species nested within *Rhabdophis*, as sister to *R. himalayanus*, and despite stating in the text (p. 21) that they declined to synonymize the genera, they recognized the species as *R. ceylonensis* in their figure 7a. Our analysis also strongly supports a sister relationship between *B. ceylonensis* and *R. himalayanus*, and thus, we formally propose that *Balanophis* be synonymized with *Rhabdophis*.

Our analysis includes three of the four currently recognized species of *Macropisthodon* (Wallach et al., 2014), no two of which are recovered as each other's closest relative. When the genus was described by Boulenger (1893), most other natricine snakes were treated as members of the genus *Tropidonotus*. Stejneger (1907) placed *Tropidonotus* in the genus *Natrix*, where it remained until Malnate (1960) divided *Natrix* sensu lato into six genera, resurrecting *Rhabdophis* Fitzinger, 1843. Malnate suggested that *Macropisthodon* might later prove not to be distinct from *Rhabdophis*, but the overreliance on characters of the maxillary dentition had precluded its earlier inclusion in *Natrix* and presumably influenced Malnate's decision to retain the genus. In our analysis, the type species of *Macropisthodon*,* M. flaviceps*, is strongly supported as sister to *R. murudensis*. Figueroa et al. (2016) show the fourth species, *M. rhodomelas*, nested well within *Rhabdophis*. Therefore, we synonymize *Macropisthodon* with *Rhabdophis*. Thus, it is presently reasonable to include all species belonging to the NGC within *Rhabdophis*, the type species of which is *R. subminiatus*. However, partitioning of this morphologically diverse clade should be considered in the future.

The divergent position of *Macropisthodon rudis*, which lacks nuchal glands and is recovered as distant from the NGC, supports the resurrection of the monotypic genus *Pseudoagkistrodon* (Van Denburgh 1909), as suggested by Wallach et al. (2014). Although recent studies have differed in the exact placement of this species (Guo et al., 2012, 2014), no analysis with sufficient taxon sampling of Asian natricines has placed it close to *Rhabdophis*. The taxonomic status of “*R”. conspicillatus* and *“R”. chrysargos*, which lie just outside the NGC, remains to be determined.

Our analysis suggests that *Rhabdophis* contains several undescribed species. Substantial genetic divergence occurs within *R. nigrocinctus, R. swinhonis, R. nuchalis*, and especially *R. subminiatus*. A comprehensive analysis of this complex subclade, including both morphological and molecular studies, will be necessary before this group can be reliably partitioned.

## 5 CONCLUSIONS

Our analysis indicates that the nuchal and nucho‐dorsal glands, as a group, have evolved only once among Asian natricine snakes. The absence of the nuchal glands in some nominally congeneric species, such as *M. rudis, R. conspicillatus,* and *R. chrysargos*, reflects old classifications based on phenetic analysis of morphological characters. All of those species lie outside the single clade that possesses the defensive glands. However, a few species within the nuchal gland clade (*M. flaviceps, R. murudensis,* and *R. swinhonis*) may represent a transitional stage in the secondary loss of the glands. Clarification of the developmental origin of these unique organs is likely to provide insight into how these neomorphic structures have arisen, diversified, and may subsequently be disappearing in a few species. The nuchal glands are fruitful subjects for investigating the evolution of novel biological systems that involve the complex interplay of morphology, physiology, ecology, and behavior.

## AUTHOR CONTRIBUTIONS

Hirohiko Takeuchi designed and performed research, analyzed data, and wrote the paper. Alan H. Savitzky designed research and wrote the paper. Li Ding designed and performed research in China. Anslem de Silva performed research in Sri Lanka. Indraneil Das performed research in Malaysia. Tao Thien Nguyen performed research in Vietnam. Tein‐Shun Tsai performed research in Taiwan. Teppei Jono performed research in China and analyzed data in Japan. Guang‐Xiang Zhu performed research in China. Dharshani Mahaulpatha performed research in Sri Lanka. Yezhong Tang designed and performed research in China. Akira Mori designed and performed research and wrote the paper.

## DATA ACCESSIBILITY

DDBJ accessions LC325298–LC325357, LC325746–LC325803, and LC326011–LC326031 (DNA sequences).

## APPENDIX 1

### 1.1 Accession numbers and their localities (countries) for all DNA sequence data used in the phylogenetic analyses in this study. Individuals with an asterisk indicate identical sequences within the species, and thus have the same accession number. Names (and No.) in the species column correspond to those shown in Figure 1

**Table:**

Species	Individual No.	Country	Accession no. of GenBank			References
			Cyt.b	C‐mos	Rag‐1	
*Afronatrix anoscopus*	ROM19842	Liberia	AF420073	AF471123	EU402832	Lawson et al., 2005, de Queiroz, Lawson, and Lemos‐Espinal 2002, and Wiens et al. (2008)
*Amphiesma stolatum*_1	HT0548	China	LC325319	LC325765	–	This study
*Amphiesma stolatum*_2	HT0798	Sri Lanka	LC325347	LC325793	LC326030	This study
*Amphiesma stolatum*_3	GP2213	China	KJ685693	KJ685643	KJ685585	Guo et al. (2014)
*Aspidura guentheri*	RAP0437	Sri Lanka	KC347472	KC347380	KC347418	Pyron, Kandambi et al. (2013)
*Atretium schistosum*_1	HT0799	Sri Lanka	LC325348	LC325794	–	This study
*Atretium schistosum*_2	–	Sri Lanka	KC347487	KC347383	KC347421	Pyron Kandambi et al. (2013)
*Atretium yunnanensis*	GP842	China	JQ678448	JQ281787	KJ685602	Guo et al. (2014)
*Balanophis ceylonensis*_1	HT0785	Sri Lanka	LC325339	LC325785	LC326026	This study
*Balanophis ceylonensis**_2	HT0786	Sri Lanka	LC325339	–	–	This study
*Balanophis ceylonensis*_3	HT0787	Sri Lanka	LC325340	LC325786	–	This study
*Haplocercus ceylonensis*	RS145	Sri Lanka	KC347478	KC347401	KC347438	Pyron, Kandambi et al. (2013)
*Hebius atemporale*	HT0550	China	LC325320	LC325766	–	This study
*Hebius craspedogaster*	HT0801	China	LC325350	LC325796	–	This study
*Hebius ishigakiensis*	HT0800	Japan	LC325349	LC325795	–	This study
*Hebius khasiense*	HT0679	Vietnam	LC325327	LC325773	–	This study
*Hebius octolineatus*	HT0586	China	LC325321	LC325767	–	This study
*Hebius pryeri*	HT0340	Japan	LC325312	LC325758	–	This study
*Hebius vibakari*_1	HT0274	Japan	LC325309	LC325755	–	This study
*Hebius vibakari*_2	HT0277	Japan	LC325310	LC325756	–	This study
*Macropisthodon flaviceps*	HT0809	Malaysia	LC325355	LC325801	–	This study
*Macropisthodon plumbicolor*_1	HT0782	Sri Lanka	LC325336	LC325782	LC326025	This study
*Macropisthodon plumbicolor*_2	HT0783	Sri Lanka	LC325337	LC325783	–	This study
*Macropisthodon plumbicolor*_3	HT0784	Sri Lanka	LC325338	LC325784	–	This study
*Macropisthodon rudis*_1	HT0339	China	LC325311	LC325757	LC326016	This study
*Macropisthodon rudis*_2	GP1266	China	JQ687452	JQ687434	KJ685566	Guo et al. (2014)
*Natriciteres olivacea*	–	Congo	AF471058	AF471146	–	Lawson et al. (2005)
*Natrix maura*_1	–	Spain	AY866530	–	–	Guicking, Lawson, Joger and Wink (2006)
*Natrix maura*_2	–	Tunisia	AY487682	–	–	Guicking, Joger and Wink (2008)
*Natrix maura*_3	–	Italy	AY487683	–	–	Guicking et al. (2008)
*Natrix natrix*_1	–	Spain	AY866536	–	–	Guicking et al. (2006)
*Natrix natrix*_2	–	France	AY866537	–	–	Guicking et al. (2006)
*Natrix tessellata*_1	–	Iran	AY487574	–	–	Guicking et al. (2006)
*Natrix tessellata*_2	–	Iran	AY487575	–	–	Guicking, Joger and Wink (2009)
*Natrix tessellata*_3	–	Bulgaria	AY866533	–	–	Guicking et al. (2006)
*Nerodia cyclopion*	–	USA	AF402909	–	–	Alfaro and Arnold (2001)
*Nerodia erythrogaster*	–	USA	AF402912	–	–	Alfaro and Arnold (2001)
*Nerodia fasciata*	–	USA	AF402910	–	–	Alfaro and Arnold (2001)
*Nerodia floridana*	–	USA	AF402911	–	–	Alfaro and Arnold (2001)
*Nerodia rhombifer*	–	USA	AF402915	–	–	Alfaro and Arnold (2001)
*Nerodia sipedon*	–	USA	AF402913	–	–	Alfaro and Arnold (2001)
*Nerodia taxispilota*	–	USA	AF402914	–	–	Alfaro and Arnold (2001)
*Opisthotropis cheni*	GP383	China	GQ281779	JQ687441	KJ685595	Guo et al. (2012)
*Opisthotropis guangxiensis*	GP746	China	GQ281776	JQ687447	–	Guo et al. (2012)
*Opisthotropis lateralis*	GP646	China	GQ281782	JQ687445	–	Guo et al. (2012)
*Opisthotropis latouchii*	GP647	China	GQ281783	JQ687446	–	Guo et al. (2012)
*Opisthotropis typica*	HT0794	Malaysia	LC325343	LC325789	LC326028	This study
*Pseudoxenodon macrops* (Out group)_1	HT0646	China	LC325323	LC325769	–	This study
*Pseudoxenodon macrops* (Out group)_2	HT0802	Malaysia	LC325351	LC325797	–	This study
*Regina grahami*	–	USA	AF402918	–	–	Alfaro and Arnold (2001)
*Regina rigida*_1	–	USA	AF402919	–	–	Alfaro and Arnold (2001)
*Regina rigida*_2	CAS:HERP:165994	USA	AF471052	AF471120	–	Lawson et al. (2005)
*Regina septemvittata*	–	USA	AF402917	–	–	Alfaro and Arnold (2001)
*Rhabdophis adleri*_1	HT0831	China	LC325356	LC325802	–	This study
*Rhabdophis adleri*_2	HT0832	China	LC325357	LC325803	–	This study
*Rhabdophis callichromus*_1	HT0654	Vietnam	LC325324	LC325770	–	This study
*Rhabdophis callichromus*_2	HT0674	Vietnam	LC325325	LC325771	LC326020	This study
*Rhabdophis chrysargos*	HT0342	Malaysia	LC325313	LC325759	LC326017	This study
*Rhabdophis conspicilatus*	HT0791	Malaysia	LC325342	LC325788	LC326027	This study
*Rhabdophis formosanus*_1	HT0033	Taiwan	LC325304	LC325750	–	This study
*Rhabdophis formosanus**_2	HT0031	Taiwan	LC325304	–	–	This study
*Rhabdophis formosanus**_3	HT0030	Taiwan	LC325304	–	–	This study
*Rhabdophis guangdongensis*	SYSr000018	China	KF800930	KF800920	–	Zhu et al. (2014)
*Rhabdophis himalayanus*_1	HT0847	China	LC325299	LC325746	LC326011	This study
*Rhabdophis himalayanus**_2	HT0848	China	LC325299	–	–	This study
*Rhabdophis himalayanus**_3	HT0849	China	LC325299	–	–	This study
*Rhabdophis himalayanus*_4	CAS224420	Myanmar	KF800929	KF800919	–	Zhu et al. (2014)
*Rhabdophis lateralis*_1	HT0855	China	LC325302	–	–	This study
*Rhabdophis lateralis*_2	GP613	China	JQ687444	GQ281785	KJ685600	Guo et al. (2014)
*Rhabdophis leonardi*_1	HT0851	China	LC325300	LC325747	LC326012	This study
*Rhabdophis leonardi*_*2	HT0852	China	LC325300	–	–	This study
*Rhabdophis leonardi*_*3	HT0853	China	LC325300	–	–	This study
*Rhabdophis leonardi*_4	RDQ200905367	China	KF800932	KF800922	–	Zhu et al. (2014)
*Rhabdophis murudensis*	HT0788	Malaysia	LC325341	LC325787	–	This study
*Rhabdophis nigrocinctus*_1	HT0253	Thailand	LC325307	LC325753	LC326015	This study
*Rhabdophis nigrocinctus*_2	HT0343	Thailand	LC325314	LC325760	–	This study
*Rhabdophis nigrocinctus*_3	HT0845	China	LC325298	–	–	This study
*Rhabdophis nuchalis*_1	HT0701	China	LC325333	LC325779	LC326022	This study
*Rhabdophis nuchalis*_2	HT0803	China	LC325352	LC325798	–	This study
*Rhabdophis nuchalis*_3	HT0807	China	LC325353	LC325799	LC326031	This study
*Rhabdophis nuchalis*_4	HT0854	China	LC325301	LC325748	–	This study
*Rhabdophis nuchalis*_5	SICAU090001	China	KF800925	KF800935	–	Zhu et al. (2014)
*Rhabdophis pentasupralabialis*_1	HT0699	China	LC325331	LC325777	–	This study
*Rhabdophis pentasupralabialis*_2	HT0700	China	LC325332	LC325778	LC326021	This study
*Rhabdophis pentasupralabialis*_3	HT0808	China	LC325354	LC325800	–	This study
*Rhabdophis subminiatus*_1	HT0267	Laos	LC325308	LC325754	–	This study
*Rhabdophis subminiatus*_2	HT0344	Thailand	LC325315	LC325761	–	This study
*Rhabdophis subminiatus*_3	HT0345	Thailand	LC325316	LC325762	–	This study
*Rhabdophis subminiatus*_4	HT0680	Vietnam	LC325328	LC325774	–	This study
*Rhabdophis swinhonis*_1	HT0021	Taiwan	LC325303	LC325749	–	This study
*Rhabdophis swinhonis*_2	HT0717	Taiwan	LC325334	LC325780	LC326023	This study
*Rhabdophis swinhonis**_3	HT0716	Taiwan	LC325334	–	–	This study
*Rhabdophis swinhonis**_4	HT0718	Taiwan	LC325334	–	–	This study
*Rhabdophis swinhonis**_5	HT0719	Taiwan	LC325334	–	–	This study
*Rhabdophis tigrinus*_1	HT0098	Japan	LC325305	LC325751	LC326013	This study
*Rhabdophis tigrinus*_2	HT0177	Japan	LC325306	LC325752	LC326014	This study
*Sibynophis subpunctatus* (Out group)	RAP0491	Sri Lanka	KC347471	KC347411	KC347449	Pyron, Kandambi et al. (2013)
*Sinonatrix aequifasciata*_1	HT0678	Vietnam	LC325326	LC325772	–	This study
*Sinonatrix aequifasciata*_2	HT0681	Vietnam	LC325329	LC325775	–	This study
*Sinonatrix aequifasciata*_3	GP357	China	JQ687430	JQ687440	–	Guo et al. (2012)
*Sinonatrix annularis*	GP889	China	JQ687431	JQ687449	KJ685604	Guo et al. (2012, 2014)
*Sinonatrix percarinata*	GP956	China	JQ687433	JQ687451	KJ685607	Guo et al. (2012, 2014)
*Storeria dekayi*	CAS:HERP:196039	USA	AF471050	AF471154	–	Lawson et al. (2005)
*Thamnophis butleri*	–	USA	AF402923	–	–	Alfaro and Arnold (2001)
*Thamnophis couchii*	–	USA	AF402936	–	–	Alfaro and Arnold (2001)
*Thamnophis cyrtopsis*	–	USA	AF402924	–	–	Alfaro and Arnold (2001)
*Thamnophis elegans*	–	USA	AF402925	–	–	Alfaro and Arnold (2001)
*Thamnophis godmani*	–	Mexico	AF420135	–	–	Alfaro and Arnold (2001)
*Thamnophis marcianus*	–	USA	AF402926	–	–	Alfaro and Arnold (2001)
*Thamnophis ordinoides*	–	USA	AF402927	–	–	Alfaro and Arnold (2001)
*Thamnophis proximus*	–	–	AF402928	–	–	Alfaro and Arnold (2001)
*Thamnophis radix*	–	USA	AF402934	–	–	Alfaro and Arnold (2001)
*Thamnophis sirtalis*_1	–	–	AF402929	–	–	Alfaro and Arnold (2001)
*Thamnophis sirtalis*_2	–	–	AF402930	–	–	Alfaro and Arnold (2001)
*Trachischium monticola*	GP1487	China	JQ687428	JQ687435	KJ685570	Guo et al. (2012, 2014)
*Xenochrophis asperrimus*_1	HT0797	Sri Lanka	LC325346	LC325792	–	This study
*Xenochrophis asperrimus*_2	–	Sri Lanka	KC347480	KC347414	KC347451	Pyron, Kandambi et al. (2013)
*Xenochrophis flavipunctatus*	HT0682	Vietnam	LC325330	LC325776	–	This study
*Xenochrophis maculatus*	HT0720	Malaysia	LC325335	LC325781	LC326024	This study
*Xenochrophis piscator*_1	HT0347	Thailand	LC325317	LC325763	LC326018	This study
*Xenochrophis piscator*_2	HT0371	Vietnam	LC325318	LC325764	–	This study
*Xenochrophis piscator*_3	HT0796	Sri Lanka	LC325345	LC325791	–	This study
*Xenochrophis trianguligerus*	HT0795	Malaysia	LC325344	LC325790	LC326029	This study
*Xenochrophis vittatus*_1	HT0615	Indonesia	LC325322	LC325768	LC326019	This study
*Xenochrophis vittatus**_2	HT0527	Indonesia	LC325322	–	–	This study
